# Tuberculosis in Cattle*Wochenschrift für Theirheilkunde, etc., No. 6, 1884.

**Published:** 1884-07

**Authors:** 


### TUBERCULOSIS IN CATTLE.*

A paper upon this subject was lately read and very earnestly discussed before the Veterinary Society at Munich, and the following conclusions adopted:

1st. The disease is transmissible from parent to offspring; a tuberculous bull can infect the ovum of the cow during the act of coition, and congenital tuberculosis appears in the young.

(We doubt this, or else the original is not explicit enough. The cause is, the weak, sensitive tissues are congenital; but ante-partum tuberculosis is something which we are at present quite sceptical about.—ED.)

2d. The embryo can also be infected from a diseased mother.

3d. Calves that are born healthy can become tuberculous from being fed on milk derived from cows having tuberculosis.

4th. That the disease may be transmitted from one animal to another by means of cohabitation, has been proved beyond all question.

The author of the above paper draws a very fine distinction as to what is to be considered general tuberculosis in the inspection of slaughtered animals, viz:

When the disease extends "per continuitatem," that is, out from one centre of infection to parts in the immediate vicinity, or has gradually extended in this way to organs quite distant

* Wochenschrift für Theirheilkunde, etc., No. 6, 1884.

from the original seat of the disease, then it is not general tuberculosis; but where we find a centre in one point, and other centres in other glands and organs of the body distantly situated, then we are to assume that the disease has extended by means of the circulation system, and the flesh is unfitted for human food. It is well known that at present not a single case of tuberculosis in man has been directly traceable to the consumption of beef taken from animals having the kindred disease.

Notwithstanding this fact, there is no question that in many cases we are justified in excluding such meat from sale and consumption, for it cannot be pronounced to be free from disease in any sense of the word.

For the dukedom of Hesse, the Ministerium has declared "that when the disease has acquired but a slight organic extension, and when the animal has not become cachectic, then such meat may be sold when marked and branded, and the consumer is told that he is buying such meat, and that with it must go a warning that it must be well cooked before being eaten."

